# SERS Immunosensor of Array Units Surrounded by Particles: A Platform for Auxiliary Diagnosis of Hepatocellular Carcinoma

**DOI:** 10.3390/nano10102090

**Published:** 2020-10-21

**Authors:** Mingyu Cheng, Yongjun Zhang, Yaxin Wang, Aonan Zhu, Lei Chen, Zhong Hua, Xiaolong Zhang

**Affiliations:** 1 School of Material and Environmental Engineering, Hangzhou Dianzi University, Hangzhou 310012, China; chengmingyu0531@163.com (M.C.); wangyaxin1010@126.com (Y.W.); 2 Key Laboratory of Functional Materials Physics and Chemistry, Ministry of Education, College of Physics, Jilin Normal University, Changchun 130103, China; chenlei@jlnu.edu.cn (L.C.); hz196110@126.com (Z.H.); 3 College of Chemistry, Nankai University, Tianjin 300071, China; aonanzhu@126.com

**Keywords:** SERS, FDTD, units surrounded by particles, AFP-L3, HCC

## Abstract

Hepatocellular carcinoma (HCC) is one of the diseases with high mortality worldwide, so its early diagnosis and treatment have attracted much attention. Due to the advantages of the high sensitivity of surface-enhanced Raman scattering (SERS) detection, SERS has excellent application value in the diagnosis of HCC. In this paper, silver nanoparticles (AgNPs) are modified by magnetron sputtering on the surface of polystyrene (PS) templates with spheres of two different diameters. The array of units surrounded by particles is successfully prepared and the SERS performance is characterized. The effect of the gap between AgNPs on plasmon coupling and hot spot distribution is discussed. Finite-difference time domain (FDTD) simulation is used to verify the electric fields and hot spot distribution of the array. The differences in the concentrations of HCC markers are analyzed by using the change of SERS signal intensity of the array. The whole process proves that the preparation of structures with a strong local electric field to provide highly sensitive SERS signals is a key link in the detection of HCC markers, which is conducive to the diagnosis of HCC and has potential application value in clinical diagnosis.

## 1. Introduction

Hepatocellular carcinoma (HCC) is the second leading cause of cancer death worldwide, killing nearly one million people each year [1,2]. The high rate of HCC metastasis due to delayed diagnosis and the high recurrence rate in some patients within 5 years after treatment are the main reasons for the high HCC mortality [1,3]. In recent years, detection technology has greatly progressed, resulting in improved diagnosis and treatment of many diseases [4,5,6,7,8,9,10], especially in the treatment of cancer; however, the survival rate is still low due to the late diagnosis of most cancers, such as HCC. The root cause of this condition is the lack of a rapid and accurate method for early diagnosis. Therefore, some new immunoassay techniques such as antigen detection have attracted more attention [11,12,13]. In recent years, diagnostic research on HCC has mainly focused on the detection of associated biomarkers. For example, Bai et al. combined magnetism to detect and analyze three cancer markers simultaneously [11]. Yang et al. used enzyme-induced core–shell structures to detect the HCC marker α-fetoprotein (AFP) [14]. Among them, AFP is one of the biomarkers used for HCC, which can realize the value of diagnosis and prediction [15]. Moreover, the biomarker lens culinaris agglutinin-reactive of alpha-fetoprotein (AFP-L3) is produced by malignant hepatocytes at the early stage of HCC and the fucosylated form of AFP. The detection AFP-L3 is less limited than the detection of AFP, which is more conducive to the diagnosis of HCC and is more widely used in the detection of HCC [15,16]. With the aim of better understanding the causes, diagnoses, and treatments of diseases, the detection and analysis of the specificity and sensitivity of the biomolecules associated with HCC have never stopped [17]. Many detection methods of AFP-L3 have shortcomings, such as being operationally complex and having low sensitivity. For example, a liquid-phase binding assay is licensed by the US Food and Drug Administration for in vitro diagnosis of AFP-L3 and is also available clinically in Korea, Japan, and most European countries, but its detection limit is 0.3 ng/mL [18]. Therefore, the choice of a method that can reduce the detection limit of AFP-L3 should also be considered.

Because Raman spectroscopy provides fingerprint vibration information based on the composition, symmetry, and environment of a sample, it has been successfully used to measure the chemical composition [19,20], molecular structure, and other changes in the object to be tested [21,22]. Raman spectra can use light in the ultraviolet and near-infrared ranges as the source of excitation, which has positive significance for biological detection [23,24]. However, Raman spectra have the disadvantage of having weak signals, which limits the wide application of Raman spectroscopy in many fields. However, the introduction of surface-enhanced Raman scattering (SERS) observed on noble metal surfaces such as Ag greatly improved the sensitivity of the Raman spectrum [25], so the preparation of nanoarray structure with good SERS sensitivity played a crucial role in biological detection [26,27,28]. For example, Chi et al. prepared a layered lotus seed array for extraordinary surface-enhanced Raman spectra [29]. Marzan et al. contributed to the treatment and administration of cancer by using gold nanostars as the substrate and using Raman detection [30,31]. Therefore, the preparation of good SERS structures is also of great significance for accurate, simple and rapid detection of HCC markers. At present, SERS technology has become more and more recognized in biological multiple detection ability [17,24]. Since then, compared with the traditional analytical methods, SERS have unique advantages in chemistry, material science, analytical science, surface science, biomedicine, and other fields [17,32]. In particular, SERS technology made great contributions to protein identification and quantification. The new Raman displacement difference and the intensity difference of some Raman spectra become the main basis for the quantitative change [11,33]. For example, Zhao et al. used the shift of SERS spectra to detect serum MicroRNAs for the diagnosis of primary liver cancer [34]. Ma et al. conducted a quantitative analysis of HCC markers based on the changes of shift and intensity in Raman peaks [15]. However, these studies mainly focused on the detection process of markers, and lack of exploration for the optimization of substrate structure performance, but the substrate with excellent performance was also crucial for detection. In previous studies, our research group has done a lot of research on the distribution of local surface plasmon resonance (LSPR) and hot spots by preparing nanospheres [35], nanobowls [36], nanorods [22], and others arrays structure, and was able to conclude that suitable plasmon coupling played a key role in improving SERS signal.

In this study, AgNPs were sputtered on an etched template containing PS spheres with two diameters to participate in the plasmon coupling in the array of units surrounded by particles. By controlling the diameter of surrounding sphere particles, the distance between sphere particles was adjusted to increase the plasmon coupling of AgNPs and enhance the distribution of hot spots. The change of SERS peak intensity of the immunosensor was used to identify and quantify the combination of AFP-L3. The immunosensor facilitated the detection of AFP-L3, which provided an excellent method for the diagnosis of HCC. The study has an excellent development prospect and potential application value in the early diagnosis of HCC patients.

## 2. Materials and Methods

### 2.1. Materials

Polystyrene (PS) colloidal particles (10% *w*/*w*) with diameters of 100 and 500 nm were obtained from the Duke Scientific Corporation in Palo Alto, CA, USA. Anhydrous ethanol, Si wafers, hydrogen peroxide, ammonia, and sodium dodecyl sulfate were purchased from Sigma-Aldrich Co., Ltd., Beijing, China. The Ag (99.99%) target material was purchased from Beijing TIANQI Advanced Materials Co., Ltd., Beijing, China. AFP-L3 ELISA kits, phosphate-buffered saline solution (PBS; 0.01 M, pH = 7.4), and bovine serum albumin (BSA), were purchased from Beijing Dingguo Changsheng Biotechnology Co., Ltd., Beijing, China. 5,5′-Dithiobis (succinimidyl-2-nitrobenzoate) (DSNB) [15] and ultrapure water were used in the experiment.

### 2.2. Preparation of Array of Units Surrounded by Particles

The 1 × 1 cm Si wafers were placed in the solution of NH_3_·H_2_O, H_2_O_2_ and ultrapure water with a volume ratio of 1:2:6. The mixed solution was placed on a 250 °C heating table and boiled for 5 min before being stopped. The Si wafers were removed from the solution and placed into a clean beaker. Ultrapure water and alcohol were added to beakers containing Si wafers for ultrasonic cleaning three times, respectively, and the Si wafers were maintained in ultrapure water.

PS spheres with diameters of 500 and 100 nm were thoroughly mixed in the centrifuge tube at a volume ratio of 20:1. The alcohol at a volume of 130 μL was poured into the centrifuge tube and the solution was evenly mixed by ultrasound. The mixture at a volume of 50 μL was dispersed on a Si wafer that had been pre-soaked for a night in sodium lauryl sulfate solution. The Si wafers were tilted and slowly immersed in deionized water. After an orderly monolayer had formed on the surface of the water, a few drops of 2% sodium dodecyl sulfate solution were dripped onto the water in order for these PS spheres to compact. After removing the monolayer from the water surface with the cleaned Si wafers, the PS sphere templates with two different diameter sizes were prepared after drying.

The etching machine was injected with argon and the PS template obtained in the previous step was etched for 10, 20, 30, and 40 s in a high vacuum state (10^−1^ Torr). Then, the diameter of the 100 nm spheres was reduced using the above etching technology, resulting in a gap between the spheres. The SEM images of the etched PS templates are shown in Figure S1 of the Supplementary Materials. In the magnetron sputtering deposition process, the gas flow into the argon was set at 20 cfm and the Ag target was placed on the magnetic DC target and sputtered on the etched PS template at a power of 10 W for 2 min. Since the diameter of PS spheres in the array before sputtering was about 73 nm (Figure S2) and the diameter of the spheres after sputtering was about 83 nm, the Ag layer of 5 nm was formed on the surface of the etched PS spheres. Through the above steps of etching and sputtering the Ag, the structure defined as the array of units surrounded by the particles was prepared.

### 2.3. Finite-Difference Time Domain (FDTD) Simulations

The electric field distribution in the array of units surrounded by particles was simulated by FDTD. The simulated geometric parameters were derived from SEM images. The infinite zone was established with periodic boundary conditions in the *x*-axis and *y*-axis directions. There were perfectly matched layers along the *z*-axis direction. The wavelength of incident light was 633 nm and the mesh size was 1 nm. The refractive index of PS was set to 1.59. The permittivity and permeability values for Ag in the material database were used. The magnitude distribution of the electric field was *|E*/*E*_0_*|* in the frequency domain field monitor under plane-wave polarized light. The simulation method had high precision and was suitable for all kinds of complex material structures.

### 2.4. Modification of HCC Markers

The DSNB acetonitrile solution was prepared at a concentration of 10^−3^ mol/L. The array of units surrounded by particles was soaked in 400 μL DSNB acetonitrile solution for 12 h to connect the DSNB probe and the substrate was rinsed with ethanol. The substrate was placed in an AFP-L3 standard antibody solution for 2 h at 37 °C, and the physically adsorbed antibodies were flushed away with PBS (PH = 7.4). The substrate was cultured in PBS solution containing 1% BSA for two hours and then exposed to AFP-L3 antigens of different concentrations (0.25, 0.5, 1, 2, and 4 ng/mL) for two hours at 37 °C. The arrays were modified with different concentrations of antigens for further SERS detection and analysis.

### 2.5. Characterization of Structure and SERS

SEM characterizations were performed on a JEOL-6500F (JEOL LTD., Tokyo, Japan) scanning electron microscope with an acceleration voltage of 15 kV. X-ray diffraction (XRD) patterns were obtained using a Rigaku D/MAX 3C X-ray diffractometer (Rigaku Corporation, Tokyo, Japan) with Cu Kα radiation. Raman spectra were measured with a Renishaw Raman system at a laser wavelength of 633 nm. The laser beam was focused on the sample at 50 × long-range objective for characterization. The signal acquisition time was set to 10 s. Ultraviolet-visible (UV-Vis) spectra were measured by a SHIMADTU ultraviolet spectrophotometer (UV-3600).

## 3. Results and Discussion

### 3.1. Morphology Characterization of the Array of Units Surrounded by Particles

**Figure 1:**
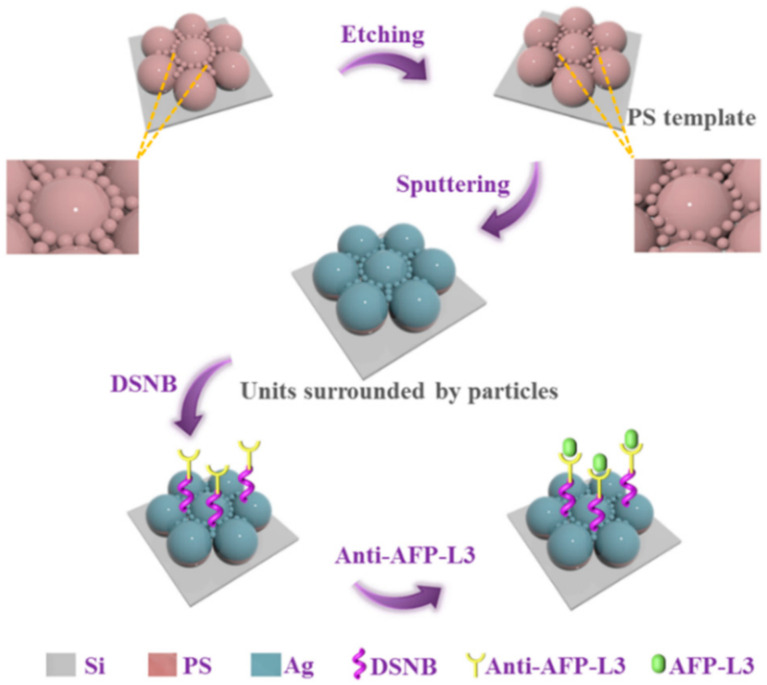
Schematic of preparation of the array of units surrounded by particles and identification of AFP-L3.

**Figure 2:**
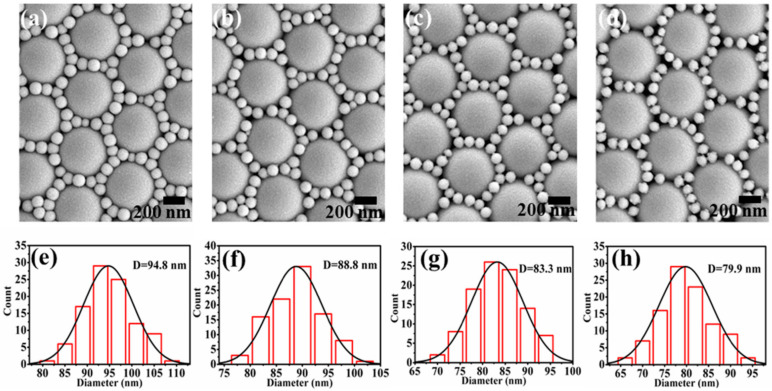
(**a**–**d**) SEM images of the array of units surrounded by particles during the PS templates, which were etched for 10, 20, 30, and 40 s. (**e**–**h**) The histograms that 100 values of the diameter of the sphere particles in the array of units surrounded by particles.

In order to enhance the SERS signal through plasmon coupling and LSPR of AgNPs on the array, spheres with diameters of 500 and 100 nm were introduced into the PS templates at the same time so that more sputtered AgNPs participated in plasmon coupling. The term “units” was used to describe the middle 500 nm spheres after sputtering of Ag, while the small PS spheres with Ag around “units” were described by the term “sphere particles”. Figure 1 shows the overall process of the experiment, in which the top half of the diagram is the preparation process of the array of units surrounded by particles. The PS templates were etched for 10, 20, 30, and 40 s before AgNPs were sputtered onto the templates’ surfaces. The SEM images of the array of units surrounded by particles obtained by characterization are shown in Figure 2a–d. With the increase of etching time, the diameter of the sphere particles around the units decreased gradually and the gap between the sphere particles increased accordingly. In Figure 2e–h, histograms were established for 100 values of the diameter of the sphere particles, which were measured in the SEM characterization images of the array of units surrounded by particles. According to the values of diameters in the histogram, the average diameters of the sphere particles were 94.8, 88.8, 83.3, and 79.9 nm. The numerical results intuitively proved the reduction in the diameter of sphere particles and the increase in gaps between sphere particles in the array of units surrounded by particles. The above differences had great impacts on the subsequent SERS performance of the structure.

### 3.2. Performance Analysis of Array of Units Surrounded by Particles

The XRD pattern of the array of units surrounded by particles shown in the Supplementary Materials (Figure S3) confirmed the presence of Ag. The wavelength of excitation light matching the absorption band of the structure was determined by the ultraviolet absorption spectrum shown in the Supplementary Materials (Figure S4). When the etching time increased to 30 s, the absorption peak at 373 nm was caused by the LSPR excitation of the regular coronal Ag layer [37]. Under the action of the electric field, the spherical cap Ag layer produced two kinds of dipole resonances, which were caused by the excitation of horizontal and vertical dipole plasmon resonances in the semi-shell [38]. Therefore, the absorption peak at 477 nm and the wide absorption band ranging 600–700 nm were easily identified, which determined the use of a 633 nm excitation line in the next SERS characterization.

**Figure 3:**
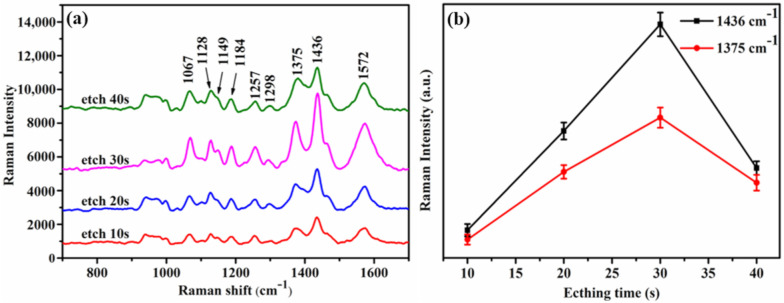
(**a**) The Raman characterization of the DSNB probe adsorbed on the array of units surrounded by particles as the etching time increased from 10 to 40 s. (**b**) The etching time of PS templates affected the variation of the peak intensities at 1436 cm^−1^ and 1375 cm^−1^. The error bars indicate the standard deviations from four measurements of the same sample.

In order to effectively improve the SERS activity, DSNB was used as the probe molecule and the array of units surrounded by particles was characterized by excitation light under the wavelength of 633 nm. Under light excitation at a fixed wavelength, the gap in the structure played an important role in the enhancement effect of SERS. The appropriate gap made a better match between the excitation wavelength and the plasmon resonance wavelength, thus the SERS signal was enhanced [39,40]. The performance of the array of units surrounded by particles with different size gaps was evaluated by SERS characterization, as shown in Figure 3a. Figure 3b also visually displays the changes of peak intensities at 1436 cm^−1^ and 1375 cm^−1^. The SERS characteristic peak intensity increased when the etching time of the PS templates increased from 10 to 30 s. The gap between the AgNPs enhanced the plasmon coupling effect and made the vibration wavelength of the surface plasmon better match the excitation wavelength to generate LSPR, caused more electric fields to be generated around this area, and generated numerous hot spots, which enhanced the SERS activity [33,41]. Therefore, as the etching time increased and the distance between the spheres increased, the plasmon coupling of adjacent AgNPs on the array of units surrounded by particles was enhanced and led to the increase in SERS intensity. However, as the etching time of PS templates reached 40 s and the distances around sphere particles gradually increased, the plasmon coupling between AgNPs was affected, resulting in a significant decrease of the electric fields and a reduction of the hot spot distribution. Therefore, as the etching time of the PS templates increased to 40 s, the radius of the sphere particles decreased to 79.9 nm after sputtering; the excessive distance might be the reason for the significant decrease of the peak intensity. The excellent repeatability and stability of the SERS signal on the array of units surrounded by particles are shown in Figure S5.

### 3.3. Finite-Difference Time Domain Simulations

**Figure 4:**
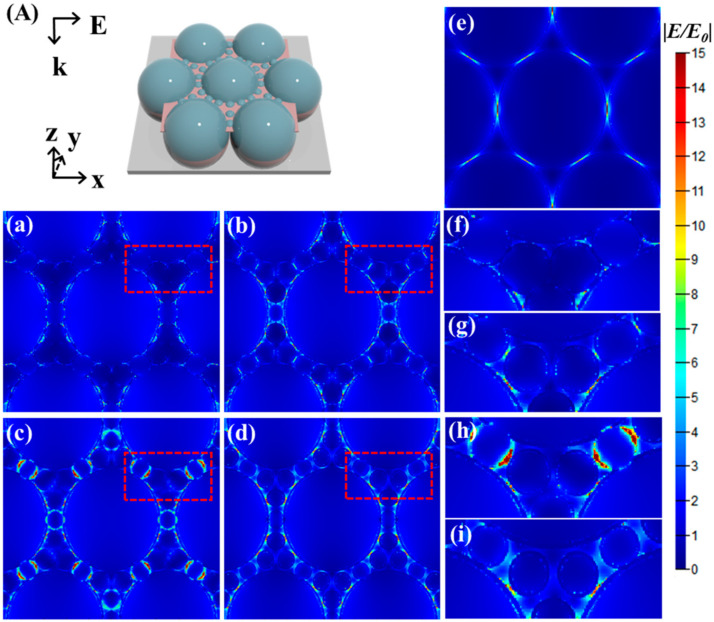
(**A**) Idealized morphology of the array of units surrounded by particles and the lattice calculation area for the periodic structure. (**a**–**d**) The localized electric field magnitude and hot spot distribution of the array of units surrounded by particles. (**e**) The localized electric field and hot spot distribution of 500 nm sphere arrays after sputtering the AgNPs. (**f**–**i**) The magnified images of the distribution of electric fields and hot spots between the sphere particles were obtained during the simulation.

The SERS characterization results obtained in the experiment were validated by FDTD simulation. The ideal shape of the array in the simulation and the horizontal cross-section minimum computational area of the periodic structures are shown in Figure 4A. The distribution of electric fields and hot spots of the array of units surrounded by particles is shown in Figure 4a–d, and the hot spot distribution of the structure obtained by sputtering Ag after 30 s of etching was higher than that of other arrays. In Figure 4f–i, the clear hot spot distribution of the regions circled in Figure 4a–d can be observed. Compared with other arrays, there are more hot spots in the gaps around the sphere particles in Figure 4h, which was the result of the strong coupling effect of AgNPs between adjacent sphere particles, meaning that the SERS signal is greatly enhanced in Figure 3a. There were also hot spots in the gap between the units and the sphere particles. However, due to the excessive etching time used for the PS spheres, the gap between the spheres after sputtering Ag became larger, which reduced the coupling between AgNPs. The above reasons led to the decrease of hot spot distribution in Figure 4d and the SERS signal in Figure 3a. In order to more intuitively show the SERS signal enhancement capability of the structure, an array containing PS spheres measuring 500 nm with Ag on the surface were also simulated and compared with the array of units surrounded by particles, the results for which are shown in Figure 4e. Compared with Figure 4c,e, there were hot spots between the units in the two structures, but there were stronger hot spots between the sphere particles in the array of units surrounded by particles, which was consistent with the ideal. The FDTD simulation results confirmed that the appropriate gaps and the high-density hot spots formed by specific structures produce stronger local electric fields, which was consistent with the SERS characterization results in the experiment [42].

From what has been discussed above, the plasmon coupling and LSPR promoted by the gap between AgNPs was the key to the electric fields causing numerous hot spots in the gap. The connection between the unit and sphere particles also contained hot spots, which enabled an excellent SERS signal in the structure. Therefore, the characteristics of the array of units surrounded by particles effectively enhanced the SERS signal and facilitated the subsequent detection of HCC markers.

### 3.4. Identification and Analysis of HCC Markers

The array of units surrounded by particles with the best SERS performance was used to analyze the concentration change of the HCC marker AFP-L3. All immunochips used for AFP detection had the same batch array to increase the rigor of the experimental process. The connection process between AFP-L3 and the array of units surrounded by particles is shown in Figure 1.

**Figure 5:**
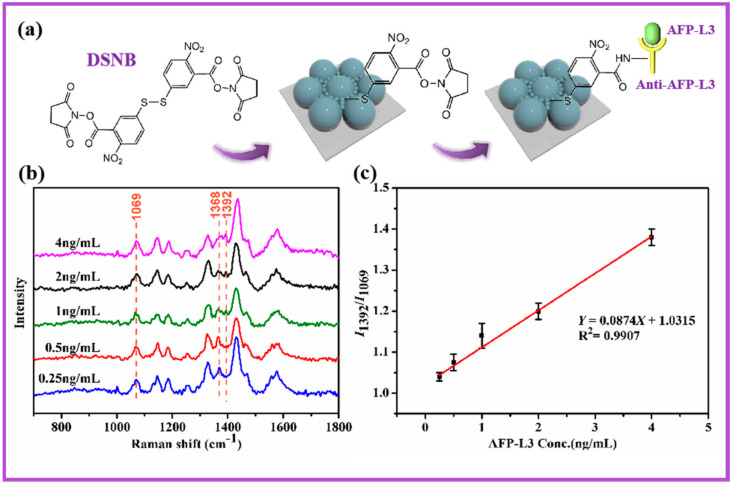
(**a**) Process analysis of AFP-L3 detected by the array of units surrounded by particles. (**b**) SERS spectra of arrays with different AFP-L3 concentration samples. (**c**) The fitting relationship between the *I*_1392_/*I*_1069_ ratio and the concentration of AFP-L3. The error bars show the standard deviations from four measurements on a sample with the same concentration.

Figure 5a shows the chemical structure of DSNB and the change of chemical bonds when AFP-L3 was connected to the array through DSNB. The Ag–S bond was formed between the DSNB and the Ag, which caused the DSNB to adsorb on the array. Additionally, the AFP-L3 antibody had an amine group (–NH_2_) that could bind to the carboxyl group (–COOH) in the DSNB molecule through dehydration and condensation reaction to form the peptide bond (–CO–NH–) in order to make the AFP-L3 antibody connected to the DSNB. With the addition of the detected AFP-L3 antigen, the peak intensity decreased significantly at 1368 cm^−1^ and increased gradually at 1392 cm^−1^. The above phenomenon was due to the AFP-L3 antigen being specifically recognized by the AFP-L3 antibody so as to be attached to the array. After the antigen was connected to the array, the Raman peak of NH–CO mode appeared at 1392 cm^−1^ and the peak intensity gradually increased with the increase of the AFP-L3 antigen concentration. The peak at 1368 cm^−1^ belonged to the COO– stretching mode, which disappeared when it was connected with biological macromolecules such as antibodies, meaning the SERS peak intensity gradually weakened [15]. Figure 5b shows the SERS characterization results of the array after connecting different concentrations (0.25, 0.5, 1, 2, 4 ng/mL) of AFP-L3 antigen. The peak at 1069 cm^−1^ with the unchanged peak intensity was selected as the internal standard peak to accurately explore the influence of AFP-L3 concentration on SERS performance. In Figure 5c, the peak intensity ratio of the array connected with different concentrations of antigen was *I*_1392_**/***I*_1069_, which had a good linear relationship. According to the fitting equation *Y* = 0.0874*X* + 1.0315, R^2^ = 0.9907, the relationship between *X* and *Y* was obtained when the peak intensity ratio of SERS was *Y* and the concentration of AFP-L3 antigen was *X*. Thus, the identification and detection of HCC marker AFP-L3 by the array of units surrounded by particles was completed.

The above detection process proved that the prepared array of units surrounded by particles could be used to analyze the HCC marker AFP-L3 and verified the feasibility and good research prospects of SERS technology.

## 4. Conclusions

In conclusion, the array of units surrounded by particles showed excellent performance in terms of SERS and the detection of AFP-L3. The experiment and FDTD simulation results showed that the appropriate gap between AgNPs could generate more hot spots in the array of units surrounded by particles, which resulted in an outstanding enhanced SERS effect, good repeatability, and excellent quantitative detection ability. The HCC marker AFP-L3 was identified and detected by the change of SERS signal intensity, so that the detection limit reached 0.25 ng/mL. This study has excellent development prospects and potential application value in the early diagnosis of HCC.

## Supplementary Materials

The following are available online at https://www.mdpi.com/2079-4991/10/10/2090/s1: Figure S1. SEM images of PS templates etched for 10, 20, 30, and 40 s. Figure S2. Histogram of the sphere diameters surrounding the unit in the PS template etched for 30 s. Figure S3. The XRD pattern of the Ag array of units surrounded by particles. Figure S4. Absorption spectra of the Ag array of units surrounded by particles. Figure S5. Characterization of the SERS stability of the array of units surrounded by particles.
