# Supplementary material for: SERS Immunosensor of Array Units Surrounded by Particles: A Platform for Auxiliary Diagnosis of Hepatocellular Carcinoma
Source: Nanomaterials (Basel). 2020 Oct 21;10(10):2090. doi: 10.3390/nano10102090 (PMC7589698; doi:10.3390/nano10102090)
Supplement: Supplementary file 1 [file nanomaterials-10-02090-s001.pdf]

nanomaterials

Supplementary Materials

SERS Immunosensor of Array Units Surrounded by Particles: A Platform for Auxiliary Diagnosis of Hepatocellular Carcinoma

Mingyu Cheng [1,2], Yongjun Zhang [1,*], Yaxin Wang [1], Aonan Zhu [3], Lei Chen [2], Zhong Hua [2] and Xiaolong Zhang [2,*]

1 School of Material and Environmental Engineering, Hangzhou Dianzi University, Hangzhou 310012, China; chengmingyu0531@163.com (M.C.); wangyaxin1010@126.com (Y.W.)
2 Key Laboratory of Functional Materials Physics and Chemistry, Ministry of Education, College of Physics, Jilin Normal University, Changchun 130103, China; chenlei@jlnu.edu.cn (L.C.); hz196110@126.com (Z.H.)
3 College of Chemistry, Nankai University, Tianjin 300071, China; aonanzhu@126.com
* Correspondence: yjzhang@hdu.edu.cn (Y.Z.); zhangxiaolong@jlnu.edu.cn (X.Z.)

Figure S1

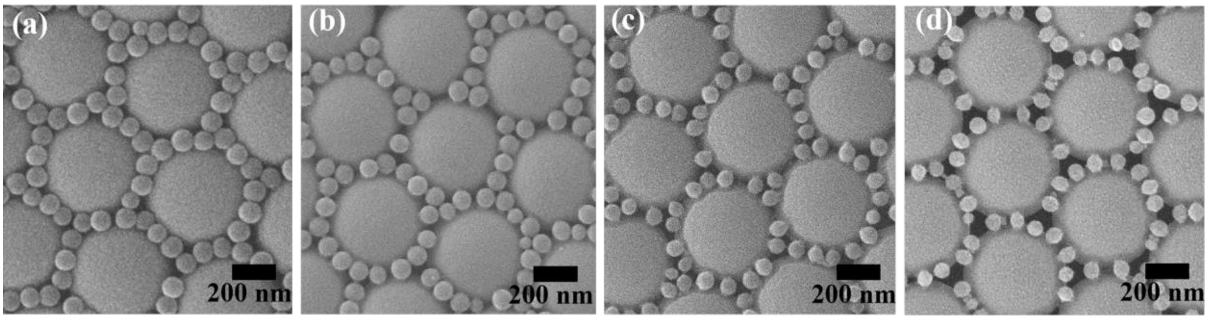

Figure S1. (**a–d**) SEM images of PS templates etched for 10, 20, 30 and 40 s.

Figure S2

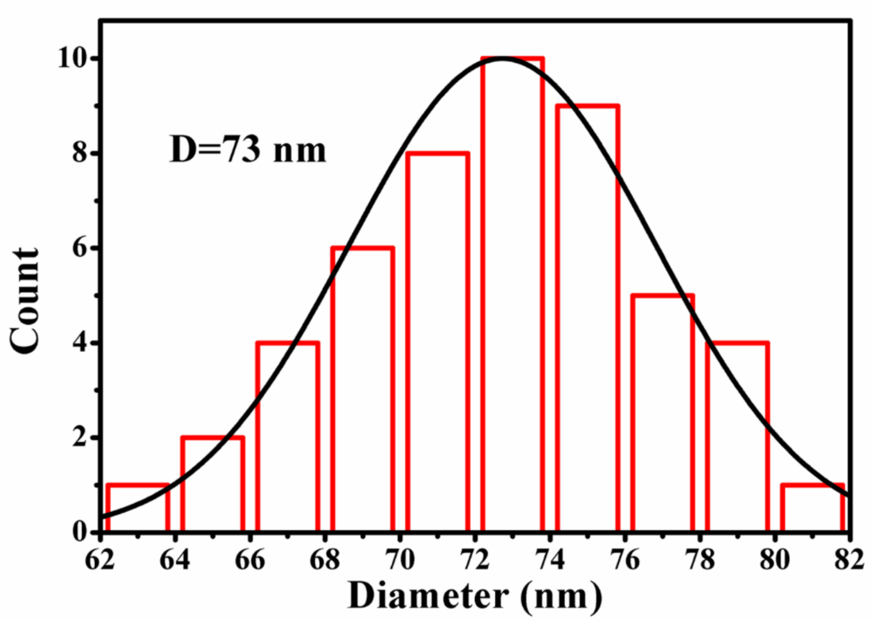

Figure S2. Histogram of the spheres diameter surrounding the unit in the PS template etched 30 s.

Figure S3

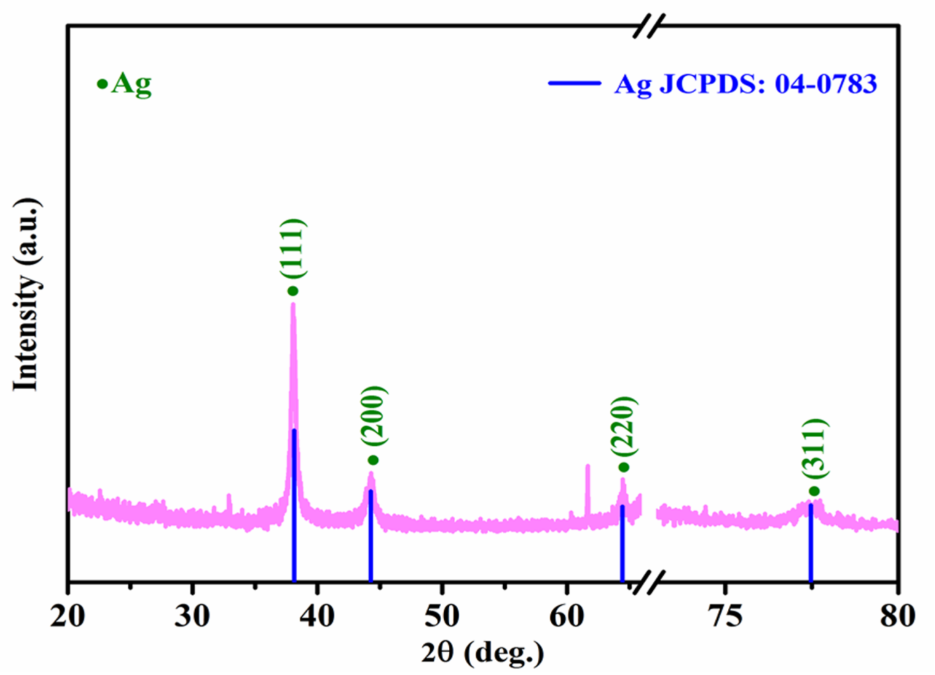

Figure S3. The XRD pattern of the Ag array of units surrounded by particles.

(111), (200), (220) and (311) crystal planes of Ag appeared. Diffraction peaks located at 37.96°, 44.2°, 64.58° and 77.26° match the corresponding plane of the Ag (JCPDS card no. 04-0783).

Figure S4

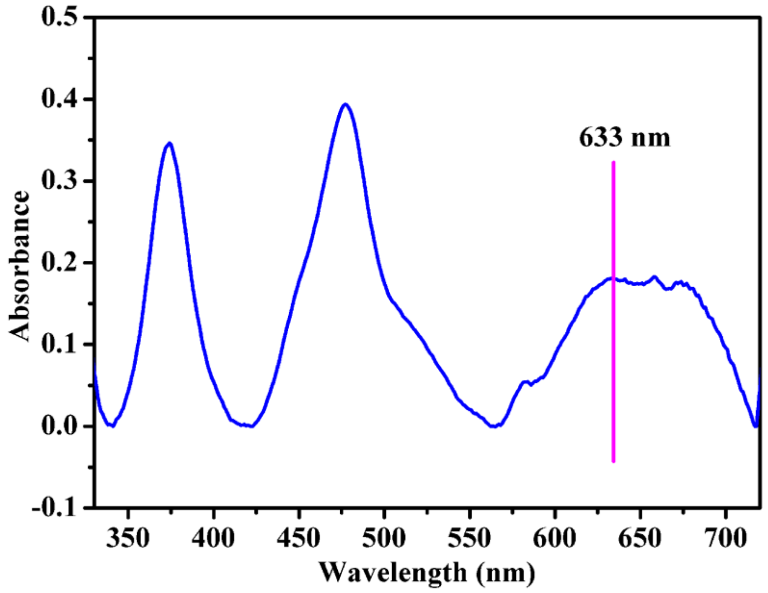

Figure S4. Absorption spectra of the Ag array of units surrounded by particles.

Figure S5

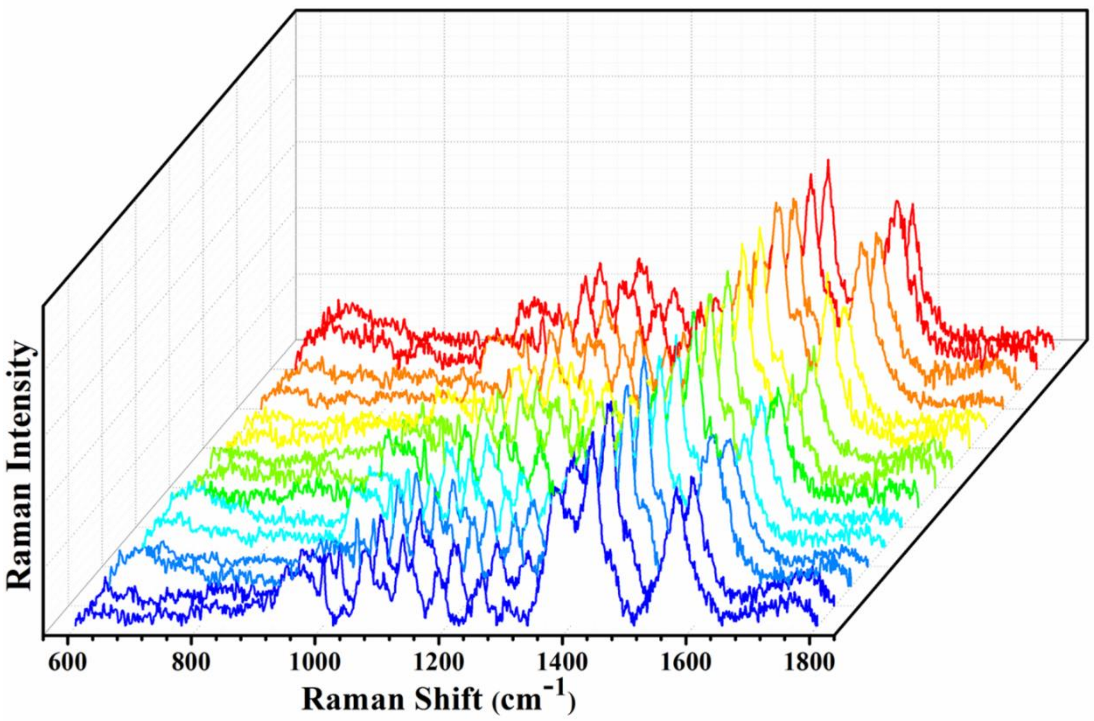

Figure S5. Characterization of the SERS stability of the array of units surrounded by particles.

The average value of the SERS peak intensity at 1436 cm^{-1}:

$$\bar{x} = \frac{\sum_{i=1}^{n} x_i}{n} = 4201.4\ cm^{-1} \quad (1)$$

The corresponding standard deviation of the peak intensity:

$$S = \sqrt{\frac{\sum_{i=1}^{n} (x_i - \bar{x})^2}{n-1}} = 132.4\ cm^{-1} \quad (2)$$
